# Book Reviews

**Published:** 1901-09

**Authors:** 


A System of Physiologic Therapeutics. A practical Exposition of the Methods, other than Drug-giving, useful in the Treatment of the Sick. Edited by Solomon Solis Cohen, A. M., M. D., Professor of Medicine and Therapeutics in the Philadelphia Polyclinic; Lecturer on Clinical Medicine at Jefferson Medical College; Physician to the Philadelphia and Rush Hospitals, etc. In eleven octavo volumes. Volume I. Electrotherapy. By George W. Jacoby, M. D., Consulting Neurologist to the German Hospital, New York City; to the Infirmary for Women and Children; and to the Craig Colony for Epileptics, etc. In two books. Book I. Electrophysics—Apparatus Required for the Therapeutic and Diagnostic use of Electricity. Book II. Diagnosis; Therapeutics. Illustrated. Philadelphia: P. Blakiston's Son & Co. 1901. [Price, eleven volumes, $22.00 net.]

This system marks a departure in the literature of therapy. It will attract attention because there is no similar series in the English language. These first two volumes of the series are devoted to the consideration of electrotherapy—the first to Apparatus and Methods; the second, to diagnosis and therapeutics. The author, George W. Jacoby, has undertaken to present all that is known in regard to machines for the generation and application of medical electricity, including all its surgical uses and radiography. A knowledge of the laws governing electrical forces is as important to the electrotherapist as to the industrial electrician. The commercial uses of the current are constantly changing and improving; the same is claimed for electricity in its medical aspects. We wish this were true and we hope it is; but so much has been claimed for it and so little has been resultant from it that our scepticism may be pardoned.

The work of this author in collecting everything of known value in regard to diagnostic therapeutics, to which the second volume is assigned, is most commendable. The student of electrotherapeutics must turn to this work for his guidance, and the specialist will need it to keep him abreast of the special applications of electricity in the treatment of disease.

The books are handsomely printed and well illustrated, the flat back style of binding, so popular in library series, having been adopted.

---

PROGRESSIVE MEDICINE, VOL. II., JUNE, 1901. A Quarterly Digest of Advances, Discoveries and Improvements in the Medical and Surgical Sciences. Edited by HOBART AMORY HARE, M. D., Professor of Therapeutics and Materia Medica in Jefferson Medical College of Philadelphia. Octavo, handsomely bound in cloth, 460 pages, with 81 engravings and one full-page plate. Issued Quarterly. Philadelphia and New York: Lea Brothers & Co. 1901. [Price, $10.00 per year.]

This volume corresponds with its predecessors as to size, style and make-up. The subjects dealt with are: Surgery of the abdomen, including hernia, by William B. Coley; Gynecology, by John G. Clark; Diseases of the blood and ductless glands, the hemorrhagic diseases, and metabolic diseases, by Alfred Stengel, and Ophthalmology, by Edward Jackson. The recent technic of various operations for radical cure of hernia are herein detailed as well as the operative treatment of gastric and intestinal diseases, while spinal anesthetisation in gynecologic operations is thoroughly set forth. An important chapter on the blood is introduced and the recent achievements of ophthalmology are recorded. The articles are published in extenso and represent the most advanced views of experts on the several subjects taken up. The illustrations are high-class and amplify the text most clearly. Progressive Medicine was awarded the grand prize at the Paris Exposition of 1900.

THE TREATMENT OF FRACTURES. By CHARLES L. SCUDDER, M. D., Assistant in Clinical and Operative Surgery, Harvard Medical School. Second edition, revised and enlarged. Octavo, 433 pages, with nearly 600 original illustrations. Philadelphia and London: W. B. Saunders & Co. 1901. [Polished Buckram, $4.50, net.]

Nine months after the appearance of the first edition of this work a second edition was published. From the start it took a high place in the literature of fractures, which it bids fair to hold for an indefinite period. In the present edition many *x*-ray plates have been reproduced which will enable the reader to interpret them without the experience of an expert; a series of clinical cases has been introduced to elaborate the chapter on fractures of the skull, and numerous illustrations have been added to the chapter on plaster-of-Paris. These are the essential changes in the second edition. It is easily one of the most helpful books that have been added lately to the literature of surgery. It deserves a place in every physician's library.

---

TRANSACTIONS OF THE AMERICAN ASSOCIATION OF OBSTETRICIANS AND GYNECOLOGISTS. Thirteenth annual meeting held at Louisville, September 18, 19 and 20, 1900. William Warren Potter, M. D, Secretary. Philadelphia: William J. Dornan, Printer. 1901.

This association has issued in this instance one of its best volumes, which is saying a good deal among so many that are good. This book is not quite as large as some of the annual transactions of this organisation but there is no padding in it, every article being of value, while some are decidedly strong in originality. Moreover, the discussions are even more forceful than ever, and these are of utmost value to both general practitioner and specialist. The illustrations, too, are among the best; by and large, therefore, we unhesitatingly pronounce this one of the most interesting and instructive of the transactions' volumes for the year. The next annual meeting of the association will be held at Cleveland, September 17, 18 and 19, 1901, as announced on another page, *q.v.*, at which Dr. William E. B. Davis, of Birmingham, Ala., will serve as president.

